# Effect combined learning on oral health self-efficacy and self-care behaviors of students: a randomized controlled trial

**DOI:** 10.1186/s12903-021-01693-y

**Published:** 2021-07-13

**Authors:** Zahra Sadat Hashemi, Mahboobeh Khorsandi, Mohsen Shamsi, Rahmatollah Moradzadeh

**Affiliations:** 1 Department of Health Education and Health Promotion, School of Health, Arak University of Medical Sciences, Arak, Iran; 2 Department of Epidemiology, School of Health, Arak University of Medical Sciences, Arak, Iran

**Keywords:** Oral and dental health, Students, Combined methods, Self-efficacy, Self-care behavior

## Abstract

**Background:**

In order to prevent oral diseases, the use of appropriate oral health education at childhood is one of the most important strategies for improving oral health knowledge and by extension positive oral health habits. Therefore, the present study aimed to evaluate the effect of animations and games as a strategy for improving oral health self-efficacy and self-care behaviors among 6–12-aged students.

**Methods:**

In this interventional study, 82 students were selected based on cluster random sampling including 38 for the case and 44 for the control group. The case group received four sessions of combined learning per week including animations and games while the control group received routine school education. The data were collected in six domains including demographics, self-care, knowledge, attitude, behavior and self-efficacy before and 5 months after the intervention using a questionnaire. SPSS version 20 was used for data analysis.

**Results:**

Five months after the intervention, the mean score of self-care, self-efficacy, behavior increased from 3.8 to 4.8, 36.8 to 48.9, and 17.07 to 18.29, respectively indicating a significant change (*p* < 0.05). However, no significant change was reported in these variables in the control group (*p * > 0.05).

**Conclusion:**

The use of animation combined with other strategies for oral health self-care education can positively influence the students' performance and self-efficacy.

*IRCT registration number* This trial was registered at IRCT. IRCT2017042133565N1 Registration date: 2017–05-17 https://en.irct.ir/trial/25851

## Background

Despite major advances in oral and dental health in many countries, oral and dental health problems are still a global challenge with a larger magnitude in populations with a weak socioeconomic status [1]. Early loss of teeth, in addition to undesirable effects on facial aesthetics and pronunciation, may result in inadequate growth and nutritional problems due to chewing difficulties. Moreover, untreated oral and dental infections have the potential to cause valvular heart disease, digestive problems, and rheumatic heart disease [2].

Based on the Iranian nation survey in 2015, the dmft index of the children aged 6 was 5.84 in a national level [1]. In addition,DMFT was identifed (5.4 ± 2.83) in Arak [2]. Further, the DMFT index of 12-year-old children was 2.09 [3]. However, the WHO emphasized that the DMFT index of 12-year-old children should be less than 1 and 90% of the 6-year-old children should have completely healthy teeth by 2010 [3].

In order to prevent oral diseases, it is necessary to implement health interventions in schools because habituation and structuring among elementary school students can make permanent and significant effects on the health behaviors of the future generation of the society. In addition, it can be considered as an effective and efficient method to transfer health messages to the students’ families and the members of society in order to promote a healthy lifestyle [4]. School children will have good dental health in adult age when they maintain this habit at school age. Thus, the pattern of regular dental attendance in early life will be continued for following years of life [5].Schools should be considered as the important places for establishing a strong link between health and education [6]. In general, health education programs are used for creating new behaviors,as well as maintaining healthy behaviors which can promote individual and community health [7]. Health education among pupil plays a signifinat role for preventing the diseases related to oral health [8].

In oral health education, we should consider the variation in dietary practices, such as controlling the consumption of cariogenic foods with a high content of carbohydrates which is important for avoiding the development of dental caries [9].Oral health education seeks to enhance oral health through educational means, especially through providing information to enhance oral health knowledge for accommodating a healthier lifestyle, and changing attitudes and behaviors [10]. Oral health behaviors and conditions have been found to be related to psychosocial factors like self-efficacy [11]. Some studies indicated the importance of the translation of the theory of self‐efficacy into the field of oral hygiene by focusing on positive relations to plaque levels, brushing frequency, and loss to periodontal follow‐up [12].

The self-efficacy refers to a person’s belief to do successfully the behavior necessary to produce the desired outcomes. It is considered as the belief related to her or his ability to do and succeed in a particular task [13]. Self-efficacy predicts a range of health behaviors including oral self-care [9]. Efficacy, by itself, has four sources including mastery experience, observing learning, verbal persuasion, and physiological and emotional states during behavioral opportunities [13]. Self-efficacy predicts a range of health behaviours including oral self-care [14]. Oral self-care behaviors are essential for promoting oral health. Self-care behaviors such as brushing teeth, flossing, and regular dental visits are recommended for oral health and gum health [15]. Moreover, the lack of self-regulatory skills is associated with a reluctance to change health behaviors, including deficits in self-efficacy, planning, and action control [9]. Studies have reported beneficial effects of self-regulatory skills on dental flossing.A combination of self-efficacy and changing oral self-care, self-efficacy, and self-monitoring planning are associated with higher frequency in dental self-care [14]. A recent study in Iran found that 25% of children never brush their teeth, indicating poor self-care in children [15].

Today in Iran,traditional health education instruments such as lectures, demonstration,and models are used in most of the strategies on promoting oral health. Based on the results, these tools play a slight or short-term effect on children [8]. In this regard, education and entertainment, such as,Games and animation, should be integrated to create a happy context for learning among children [8].

Zulkarnian [16]. indicated that cartoon animation including texts and static graphics can increase the level of KAP score more than traditional methods.Students should be exposed to dynamic images and motion pictures in the animation method.It gives them a new meaning and concept to literacy and education [17]. Furthermore, games, as a means of entertainment, can be used for education. During games, the children achieve valuable experience and learn new things willingly without pressure [18].

In self-care programs, a person is supposed to know his/her own unfavorable condition, set objectives and make plans to reach the favorable condition, become committed to execute the plan, be aware of the consequences of executing the self-care program, and learn the required skills for behavior change.According to the best of our knowledge, no program has focused on promoting self-regulation and self-efficacy, oral health with animation and game-based learning in Iran.The present study aimed to evaluate the effect of animations and games as a strategy for improving oral health self-efficacy and self-care behaviors among 6–12-aged students.

## Method

**Fig. 1:**
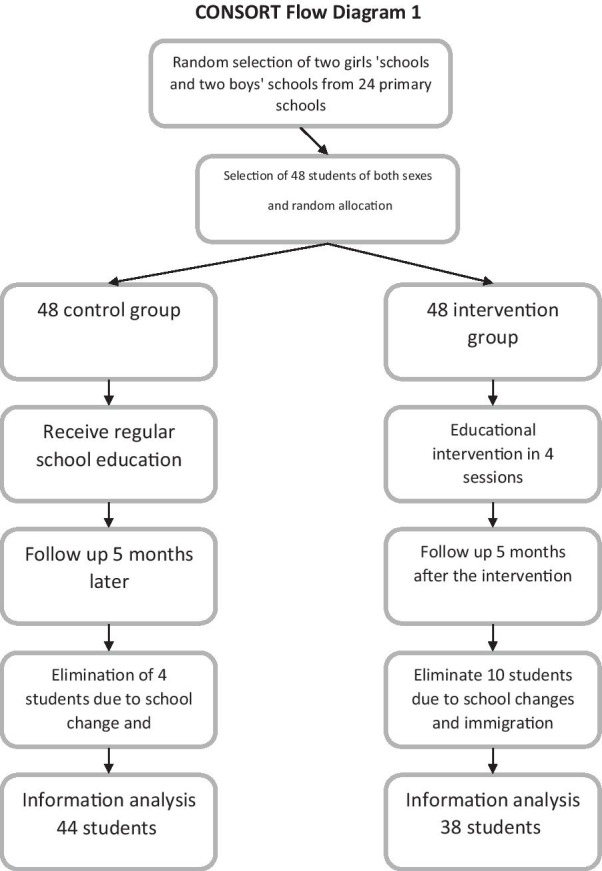
Flow of participants

The present study is an educational randomized controlled trial., we selected 82 primary school students based on multistage cluster sampling. After receiving the necessary permits to conduct research and coordinating with local authorities, two girls schools and two boys schools were randomly selected from governmental schools. Each grade was considered a cluster and 4 participants were randomly selected from each grade using class rosters. In the next stage, using simple randomization, one boy school was selected as the case school and another school was considered as the control school. The same process was applied to girl schools. The sample size, based on the results of the previous studies, was α = 0.01 and β = 0.1, and the attrition of 20% was 48 participants in the case group and 48 in the control group [19]. However, 14 students did not complete the study. Thus,the data for 82 students including 38 in the case group and 44 in the control group were considered for analysis (Fig. 1). The inclusion criteria were a willingness to participate in the study, having the consent form signed by parents,and studying in the primary schools of Khomein city. The exclusion criteria were the withdrawal from the study, missing more than one educational session, and immigration from Khomein city.

The data collection tool was a researcher-made questionnaire containing questions on self-care, knowledge, attitude, behavior, and self-efficacy,which was developed based on the questionnaires designed by Mohammadi Zeidi et al. [20] and Samiee Roudi et al. [21]. Validity of the questionnaire was assessed through content validity in two quantitative and qualitative ways and with the assistance of ten professors and specialists in health education and promotion, school health, oral and dental health, and dentists. The content validity Index and ratio of the questionnaires (CVI and CVR) was evaluated by a panel of experts including 10 experts in health education,. A CVR of above 0.62 was considered as acceptable. Regarding CVI, the experts evaluated each item in terms of relevance, clarity, and simplicity, Based on the results, all were considered as acceptable since all values were above 0.79 [22].The reliability of the questionnaire was measured through the Cronbach’s alpha coefficientin 30 students aged 6–12 years whose demographic characteristics were similar to those of the study population using the Stata 14 software [23].

The questionnaire was designed in six sections. Variables such as age, grade, gender, etc. were collected in the demographic section through the students file. The self-care section included 5 yes–no questions, and “I don’t know” as answers. The total score of this section was 5.For example, I clean my mouth and teeth myself (Yes,No, I don't know).The knowledge section contained 10 questions with three-choice answersFor example, what is the best way to clean the space between your teeth? (Toothbrush, Dental floss, Mouthwash). A score of 1 was given to correct answers, totaling a score of 10. The attitude section had 13 questions in a 5-point Likert scale from 1 to 5 with a total score of 65. For example,I think eating milk is very important for dental health (I totally agree, I agree, It doesn't matter,I disagree, I completely disagree) Behavior was assessed with 8 questions in a 5-point Likert scale with a total score of 40.For example, I wash my mouth after eating sweets and food (Always, Most of the time, Sometimes, Rarely, Never).In adition, fourteen questions were used to assess self-efficacy in a 5-point Likert scale, scoring a total of 60 points. In each of the following situations, how sure are you that you can clean your teeth? a.Not sure at all; b. a little sure; c. pretty sure; d. very sure; e. totally sure).

The structural questionnaires completed in the form of interviews to explain more details to the students and provide appropriate answers.After coordination with school principals and conducting a pretest, two schools randomly selected as the cases and received training in 4 sessions (45–60-min) (Table 1). The schools considered for control received routine school training (Educating by health Instrutor). This study caused no conflicts of interest for any person or organization.

**Table 1:** Educational content and methods of training sessions in the case group of schoolchildren

Session	Topic	Training method
One knowledge	Providing primary information about dental and oral health to students:The importance of oral hygiene, the role and use of teeth, tooth structure, number and types of teeth, how tooth decay, factors affecting dental health, ways to prevent tooth decay, how to use toothbrushes, how to use dental floss and mouthwash, time to see a dentist	Film, photographic slides, educational booklet, question and answer
Two (self-care)	Assessing oral and dental health, self-care of students: determining the objectives, setting a timetable, learning necessary skills, the importance of brushing to remove dental plaque, the definition of plaque and how tooth decay, the frequency of brushing, the duration of brushing, choosing the right toothbrush and toothpaste, how to use floss, useful and harmful foods to teeth in the form of an animation	Group discussion, lecture, animation
Three behavior	Telling students how to Brush, the use of dental floss and mouthwash, diet, healthy life style and etc. To keep this behavior going, students were asked to check their brushing teeth in the behavior chart. Then the use of dental floss was taught and a group game "Saying on the phone" was held, students were divided into two groups and placed in two rows. To the first person of each group, a sentence was said, and they were asked to slowly repeat this sentence in the next person's ear, respectively, the last person had to say the sentence completely and correctly to the instructor. Each group did this faster, won the game. In the next step the use of mouthwash was explained and the mouthwash was practically used and the animation was displayed too. Proper use of toothbrushes, floss and mouthwash was shown in 5 min with animation	Practical education, game, animation
Four self-efficacy	In addition to educational animation, games were used to practice oral and dental health skills in different situations like tiredness, disease, party, etc. Skills were learned in a variety of situations including time of fatigue, illness, lack of toothbrushes, and playing and attending a party were performed by the students themselves in a pantomime format so that the activity was performed by one student. And the other students guessed what the situation was and expressed their views on the situation. Also, the student behavior chart was reviewed a week ago, and the importance of behavior was recalled, and students were asked to find ways to recall behavior by conducting group discussions, and at the end of the discussion, repetitive topics were removed and final comments such as "Let's ask Dad and Mom to remind us" or "Put a toothbrush picture on the wall of the room." The animation was then displayed. The animation showed the importance of permanent teeth, the important of six teeth, fluoride therapy and fissure sealant, seeing a dentist and emphasizing the regular use of toothbrushes and toothpaste in 3 min. Also, it was handed over to the students to see with the family in order to repeat the lessons at home and the family's access to the educational materials of the CD animation	Animation, game, group discussion

We used the animations related to oral health that are available in Aparat.com.

Exercising and recalling the learned material during games in a simple and child-friendly atmosphere causes an emotional arousal in children. Children participated in the practical training of oral health behaviors, as a source of self-efficacy, their success in proper functioning was considered. In addition,we used positive feedback and encouragement from educators and parents as a source of verbal encouragement for children. In this study, we taught students about nutrition and its effect on oral health.Written parental informed consent, as well as written child assent, was obtained from all students participating in this study. In addition, after finishing the study, the training materials such as the booklets and CD (Animation) were given to the control group. This study was registered under the ethics code of IR.ARAKMU.REC.1395.446.

Pre and post-intervention data were collected from the children using a questionnaire( Apendix1) and entered into SPSS version 20. The questionnaires were completed in the form of structural interviews to explain more details to the students and provide appropriate answers.The Kolmogorov–Smirnov test was applied to check normal data distribution and proper statistics were used accordingly. Chi square test, paired and independent t test, Mann–Whitney U test, and Wilcoxon signed-rank test were used for statistical analysis.

## Results

The results indicated that gender, parents’ education, and number of family members had a similar distribution between the two groups (Table 2). The mean age of the subjects was 9.6 ± 1.9 years in the control group and 9.4 ± 1.8 years in the case group (Table 3). According to the independent t-test, there was no statistically significant difference in the mean scores of self-care, knowledge, attitude, behavior, and self-efficacy, score before the intervention in the study groups (p > 0.05).After the intervention in the case group, the mean scores of self-care, knowledge, attitude, behavior, and self-efficacy increased from 3.8 ± 0.96 to 4.8 ± 0.3, 6.7 ± 2.5 to 9.4 ± 0.9, 55.1 ± 12.5 to 61.5 ± 4.8, 17.07 ± 5.61 to 21.21 ± 5.07, and 36.8 ± 11.6 to 48.9 ± 10.8 respectively, which are all significant. According to paired t-test, the mean scores of self-efficacy and behavior before and after the intervention in the experimental group were significantly different (*p* < 0.05). The mean scores of attitude, knowledge and self-care before and after the intervention in the experimental group were statistically significant (p–v < 0.05). (Table 4).

**Table 2:** Frequency distribution of qualitative demographic variables in the case and control groups

Group		Case		Control		p–v
	Variable					
		Number	%	Number	%	
Father’s education	Primary school and Junior high school	7	18.4	9	20.5	0.965
	High school diploma and associate degree	19	50.00	22	50.00	
	Bachelor’s degree and higher	12	31.6	13	19.5	
Mother’s education	Primary school and Junior high school	6	15.8	11	23.3	0.458
	High school diploma and associate degree	22	57.9	26	60.5	
	Bachelor’s degree and higher	10	26.3	7	16.3	
Birth Rank	First	15	39.5	26	59.1	0.207
	Second	15	39.5	12	17.3	
	Third or higher	8	21.05	6	13.6	
Gender	Girl	22	57.9	20	45.5	0.184
	Boy	16	42.1	24	54.5

**Table 3:** Comparison of the intervention and control groups, concerning the demographic variables

	Group	Number	Mean	SD	p–v**
Number of family members	Control	44	3.93	0.9	0.533
	Case	38	4.05	0.8	
Age	Control	44	9.64	1.9	0.424
	Case	38	9.39	1.8	

*P-value > .05

**Table 4:** Mean scores of variables before and after intervention in the case and control groups

Variable	Time group	Before intervention		Five months after intervention		*p* value Wilcoxon
		Mean	SD	Mean	SD	
Self-care	Case	3.8	0.96	4.8	0.3	< 0.001
	Control	4.2	1.1	4.2	0.89	0.527
	p-value Mann Whitney	0.057	0.002			
Knowledge	Case	6.7	2.5	9.4	0.9	0.001
	Control	6.2	2.3	6.7	2.1	0.004
	p-value Mann Whitney	0.253	< 0.001			
Attitude	Case	55.1	12.5	61.5	4.8	< 0.001
	Control	54.02	14.7	55.06	11.4	0.897
	p-value Mann Whitney	0.627	< 0.001			
						*p* value paired t- test
Behavior	Case	17.07	5.61	21.21	5.07	< 0.001
	Control	18.2	5.87	17.97	5.55	0.142
	p-value t- test	0.343	0.008			
Self-efficacy	Case	36.8	11.6	48.9	10.8	< 0.001
	Control	42.11	15	41.93	14.9	0.543
	p-value t- test	0.089	0.019			

## Discussion

The present study aimed to evaluate the efficacy of combined education to promote self efficacy and self-care oral health behaviors among the students.Based on the results, no difference was observed between the intervention and control groups in terms of parents' level of education, which indicates the homogeneity of the groups.

In addition, a significant increase was observed in the mean score of self-care in the case group after the educational intervention compared to baseline, indicating the positive effect knowledge and attitude on improving self-care. The results are consistsent with those of Mohammad-Zeidi et al. [20] and Soltani, et al. [15] on the effectiveness of an educational intervention based on the stages-of-change model in improving oral health self-care behaviors among 160 male and female elementary students in Qazvin. Further, Mohammadi Zeidi et al.[24] studied the effect of motivational interviewing of oral self-care behaviors among high school students ion Qazvin and found a significant change in the mean score of behavioral intention in the case group after the intervention.In this study, health teachers were asked to teach students.The findings indicated the significance of training teachers to teach self-regulation skills in class [7].According to the National Oral Health Care Program,students follow the suit when teachers brush their teeth, which becomes a daily exercise routine. In addition, it can enhance the link between education and healthy habits since it can shape the individual’s way of life and personality. Teachers have important role and can play a major role in imparting dental health education to children by planning and implementing oral health preventive programs [5].

In this study,attempts were made to engage parents for reinforcing children oral health behavior by providing booklet and CD animation. Family and other “important others” play a role in developing self-care habits among children and young people [25].

In this study, knowledge significantly increased after the intervention in the case group, which is line with the results of the studies conducted by Goudarzi [26] and Samiee Roudi [21]. Health education is a widely accepted and practiced approach for preventing oral and dental diseases.Further, it is considered as the process which transfers the knowledge and skills required for imporving the quality of life [27]. The mean score of knowledge increased after the intervention in the control group although the difference was smaller compared to the case group. The reason for this difference could be the routine education provided by school health instructor, however this is insufficient or inadequate to help students make the transition.But Mamata Hebbal [27] found a significant change in knowledge in the control group after the intervention.The reason for the change in knowledge in the control group in our study,It may be possible that the content of the questions asked from control group have motivated them to obtain information.

The present study assessed the students’ attitude, i.e. their positive or negative thoughts about oral health behaviors. One of the assumptions in this study was a significant change in the mean score of attitude in the case group after the intervention, which was confirmed by the results. The results of Goudarzi et al. [28] and Mohd Zulkarnain Sinor et al.[16] are in line with those in the present study.

To have a healthy lifestyle, training children early forms their attitude and promotes healthy behaviors [29].

In this study, the mean score of behavior increased significantly after educational intervention by using a combination of games and animation and conducting the self-care program, compared to baseline in the intervention group, which is consistent with the results of Mohamadkhahet al. in Chabahar elementary students [24], Neha Singhet al. [30], and Goudarzi et al.[28] which reported significant changes in the score of behavior after the intervention(A 45-min educational film).

Dental caries preventive behavior is significantly and positively related with knowledge and attitude so that the increase in knowledge and attitude may be result in adopting oral health-related behaviors[10]. Moreover, having knowledge about a particular subject affects the person’s behavior in this regard [31].

Finally, the mean score of self-efficacy increased significantly in the case group after the intervention,which is consistent with the result of Mohammad-Zeidi et al. [24].

Bandora considers self-efficacy as an essential factor in changing behavior which improves through mastery over the behavior. Therefore, The self-efficacy improves with an active and successful participation in a behavior [13]. Educational interventions based on promoting self-efficacy can be useful for improving oral health behaviors in children.(11).

## Limitation

Some limitations of this study are using a self-reported questionnaires which may be prone to recall as well as desirability bias. Moreover conducting the educational intervention in all 6 primary grades, in addition to the difficulties in coordination for participation of all students, interfered with their routine classes. Due to high costs, it was not possible for the researcher to prepare the animation, and the available animations which were in accordance with the needs of the study group were used for educational intervention.Finally, it was not possible to ask a a nurse or dentist to attend the dental examination at the school, and only a check-up was taken by a health educator. Therefore, we were unable to report the dental indicators.

## Conclusion

Based on the results,the use of combined education methods including animation and game which were used to promote self-efficacy played a significant role in improving the oral health behaviors and self-efficacy in the students, in addition to improving their knowledge and changing their attitude towards the importance of oral health. The school environmenthas a great potential for educational interventions. School‑based oral health programs is more effective when education is integrated with active follow-up education for controlling and monitoring behaviors.Supporting the oral health of future generations is considered as a commitment which should be shared by parents, teachers,school administrators, and all health professionals.

Finally, instead of using traditional methods of educating students, it is recommended to use educational approaches in which studentes participate and are active. Using successful studentes as educators and using visual media in educational programs to make the training more effective are also recommended.

## Data Availability

The datasets used and.or analyzed during the current study are available upon reasonable request from the corresponding author**.**

## Abbreviations

DMFT.dmft: Decayed, missing, and filled teeth
CVR: Content validity ratio
CVI: Content validity index
WHO: World health organization
